# Chemsex as a Vector of Social Integration among Migrant Minorities: A Narrative Study

**DOI:** 10.1192/j.eurpsy.2026.10804

**Published:** 2026-07-31

**Authors:** J. Valero

**Affiliations:** 1 In hospital psychiatry, CH George Sand, Bourges; 2 Transcultural psychiatry, Université Paris Cité, Paris, France

## Abstract

**Introduction:**

*Chemsex* is defined as the use of psychoactive substances to enhance or prolong sexual encounters and has emerged as a complex socio-cultural and psychiatric phenomenon. It has become increasingly prevalent among men who have sex with men (MSM), particularly among LGBTQ+ migrant populations in urban European settings. Despite the awakened concerns in terms of public health, less attention has been paid to its symbolic and psychological role, especially for those who experience multiple layers of marginalization and the negotiation of identity within the host society.

**Objectives:**

This qualitative study explores through a narrative approach how *chemsex* practices serve as a framework for social integration among LGBTQ+ migrants, and how they intersect with the identity reconstruction and psychological vulnerability of migrant men who have sex with men (MSM).

**Methods:**

Using a phenomenological interpretive approach, two semi-structured interviews were conducted with Latin American cisgender gay men (ages 30–35) both living in France and Spain. Each participant had ongoing psychiatric treatment for anxiety disorders and reported regular engagement in *chemsex.* Interviews were thematically analysed, applying a double hermeneutic framework to uncover both individual meaning-making and structural determinants.

**Results:**

Findings reveal ambivalent experiences; *chemsex* is described as both a space of collective belonging and a space for vulnerability, dissociation, and emotional isolation. Participants identified it as a transitional space allowing sexual liberation and cultural detachment, yet also a site of addiction, trauma reenactment, and social precarity. The narratives describe a lack of culturally adapted care, highlighting difficulties in accessing adequate and non-judgmental mental health support, one that recognizes their migratory and sexual experiences accordingly to their cultural realities.

*Chemsex* appears as a paradoxical site of both integration and exclusion for LGBTQ+ migrants. It provides temporary relief from structural marginalization, while reinforcing cycles of psychological harm.

**Conclusions:**

These findings suggest that *chemsex* might not be a merely behavioural risk, but a coping strategy that reflects deeper psychosocial processes linked to migration, sexuality, and social exclusion. By framing chemsex as both a symptom and a signal, the study advocates for a more integrative model in addiction psychiatry.

**Disclosure of Interest:**

None Declared

